# Different reactions of wheat, maize, and rice plants to putrescine treatment

**DOI:** 10.1007/s12298-024-01462-5

**Published:** 2024-05-22

**Authors:** Altafur Rahman, Eszter Kulik, Imre Majláth, Imran Khan, Tibor Janda, Magda Pál

**Affiliations:** 1 Department of Plant Physiology and Metabolomics, Agricultural Institute, Centre for Agricultural Research, Hungarian Research Network, Brunszvik 2, Martonvásár, 2462 Hungary; 2 Department of Plant Physiology and Plant Ecology, Institute of Agronomy, Hungarian University of Agriculture and Life Sciences, Budapest, 1118 Hungary; 3 Syngenta Ltd., Alíz 2, Budapest, 1117 Hungary

**Keywords:** Polyamine metabolism, Oxidative stress, Reactive species, Stress markers

## Abstract

Polyamines play an important role in growth and differentiation by regulating numerous physiological and biochemical processes at the cellular level. In addition to their roborative effect, their essential role in plant stress responses has been also reported. However, the positive effect may depend on the fine-tuning of polyamine metabolism, which influences the production of free radicals and/or signalling molecules. In the present study, 0.3 mM hydroponic putrescine treatment was tested in wheat, maize, and rice in order to reveal differences in their answers and highlight the relation of these with polyamine metabolism. In the case of wheat, the chlorophyll content and the actual quantum yield increased after putrescine treatment, and no remarkable changes were detected in the stress markers, polyamine contents, or polyamine metabolism-related gene expression. Although, in maize, the actual quantum yield decreased, and the root hydrogen peroxide content increased, no other negative effect was observed after putrescine treatment due to activation of polyamine oxidases at enzyme and gene expression levels. The results also demonstrated that after putrescine treatment, rice with a higher initial polyamine content, the balance of polyamine metabolism was disrupted and a significant amount of putrescine was accumulated, accompanied by a detrimental decrease in the level of higher polyamines. These initial differences and the putrescine-induced shift in polyamine metabolism together with the terminal catabolism or back-conversion-induced release of a substantial quantity of hydrogen peroxide could contribute to oxidative stress observed in rice.

## Introduction

Polyamines (PAs) are a group of small, aliphatic organic molecules that are found widely throughout various living organisms, including plants (Tiburcio et al. 2014; Chen et al. 2019). The process of putrescine (PUT) synthesis takes place either through the decarboxylation of ornithine or indirectly via the decarboxylation of arginine (this latter reaction is catalysed by arginine decarboxylase (ADC)). The synthesis of higher PAs, namely spermidine (SPD) and spermine (SPM), involves the stepwise addition of aminopropyl moieties to the PUT structure through enzymatic reactions, catalysed by SPD synthase (SPDS) and SPM synthase. The process of catabolism in PAs is regulated by enzymes such as diamine oxidase (DAO) and PA oxidases (PAOs)*.* It is worth mentioning that two different types of PAOs exit, members of the first one play a crucial role in the final breakdown of SPD or SPM, whereas the others are involved in the partial or complete conversion of SPM to SPD and SPD to PUT. PA oxidases (PAOs) in rice have been extensively characterized regarding their subcellular distribution and temporal expression dynamics across growth stages. Notably, apoplastic PAO expression is markedly reduced during the initial two weeks following seed germination, whereas peroxisomal PAOs are prominently expressed during this pivotal developmental phase, as reported by Ono et al (2011). As a result, the PA pool demonstrates temporal variations, which are marked by swift interconversions that form the "PA cycle" (Pál et al. 2015). The collection of these polycationic compounds demonstrates a high level of precision in regulating important cellular processes, including DNA stabilization, RNA processing, maintenance of membrane integrity, and protein synthesis (Tiburcio et al. 2014). In addition to their fundamental role in normal plant development, PAs have become essential regulators in the field of plant stress adaptation. They function as molecular orchestrators that coordinate responses to various types of stress, including both abiotic and biotic factors (Pál et al. 2021; Alcázar et al. 2010; Hussain et al. 2011).

Stress tolerance in plants has been closely associated with their ability to enhance PA synthesis when exposed to stresses, often achieved through the overexpression of PA biosynthetic genes (Liu et al. 2015; Jia et al. 2021; Alcázar et al. 2020). The potential of PAs to enhance stress tolerance in crops has been suggested due to their capacity to mitigate oxidative damage resulting from environmental stressors (Takahashi and Kakehi 2009). Nevertheless, it is crucial to acknowledge that excessive accumulation of PUT during stressful circumstances, resulting in an elevated PUT/(SPD + SPM) ratio, can have adverse consequences on plant physiology (Shu et al. 2012a, b). PAs demonstrate a dual function, serving as scavengers of free radicals and as generators of free radicals (Takahashi and Kakehi 2009; Pottosin and Shabala 2014). Moreover, it should be noted that PAs also serve as signalling molecules, thereby adding complexity to their various functions (Mattoo and Sobieszczuk-Nowicka 2019). While existing research has primarily emphasized the positive effects of PAs on stress resistance, the influence of exogenous PAs on the PA pool's balance remains a critical aspect that has not been extensively discussed (Do et al. 2013; Liu et al. 2015; Shu et al. 2012a, b). Additionally, the intricate interaction between the treatment of PUT and the metabolism of PAs in various plant species continues to be a topic of research. The potential outcomes of this interaction may vary depending on the plant's initial PA levels and capacity to maintain PA balance after the supplementation of PUT. It is crucial to investigate whether the advantageous impacts of PUT treatment observed in specific crop species are universally applicable or limited to specific species.

The objective of this study is to evaluate the effect of PUT treatment on three economically important plant species, namely wheat (*Triticum aestivum*), maize (*Zea mays*), and rice (*Oryza sativa*). The hypothesis suggested that there may be variations in the response to PUT treatment among these species, which can be attributed to differences in their basal PA levels and their ability to regulate PA homeostasis. The primary objectives of the present study encompass the comparison of PA metabolism in these plants, addressing the following research inquiries: (1) Does the applied 0.3 mM PUT treatment administered over a one-week duration induce stress in rice plants, and what physiological changes (photosynthesis-related parameters are some stress markers) are evident? (2) What initial disparities in PA content exist among these plant species, and what discernible variations emerge in the PA metabolism of plants following PUT treatment, with a particular focus on significant alterations at gene expression level? (3) Are changes in PA pool predominantly influenced by the mechanism of PA uptake or the PA metabolism, and consequently responsible for the observed changes in PA pool, and in turn for the negative effects? Through systematic investigation of these three crop species' responses to PUT treatment, we aim to unravel the intricate relationship between PUT supplementation and PA metabolism.

## Material and methods

### Plant material, growth conditions, and treatment

We conducted experiments on three cereal species: wheat (*Triticum aestivum* L.), maize (*Zea mays* L.), and rice (*Oryza sativa* L.). Specifically, we chose domestically bred genotypes for each species: Béres (winter wheat variety from Martonvásár), Mv 350 (maize hybrid from Martonvásár), and Janka (japonica rice variety from Szarvas).

Rice plants have higher temperature requirements than wheat and maize, so they were grown separately. Wheat and maize were germinated between moistened filter papers at 26 °C for 3 days, with daily monitoring of germination progress, while rice seeds were placed between soaked filter papers and germinated at 37 °C for one day and then at 27 °C for 5 days, in dark.

Healthy seedlings (15 plants per beaker for wheat and rice, 6 for maize) were grown on stainless-steel nets with modified Hoagland nutrient solution (Pál et al. 2005) in a Conviron PGV-36 plant growth chamber at 22/20 °C for wheat and maize, while at 28/26 °C for rice (16/8-h light–dark cycle, 250 μmol m^−2^ s^−1^ PPFD, 75% relative humidity). After one week, maize, wheat, and rice plants were randomly divided into control (C) and PUT-treated (PUT) groups. PUT treatment was applied at 0.3 mM concentration into the nutrition solution for 7 days. This concentration and duration were chosen based on previous results on wheat, maize and rice (Szalai et al. 2017; Pál et al. 2017). During the experiment, nutrient solutions were changed every two days, and pots were randomized. After one week of PUT treatment, leaf and root samples were collected from both C and PUT-treated plants.

### Chlorophyll content measurement and chlorophyll-a fluorescence induction analysis

The youngest, completely expanded leaves were used for the measurements. The chlorophyll content was measured non-invasively with a portable SPAD-502 chlorophyll meter (Konica Minolta, Inc. Japan). The recorded values ranged from 0 to > 100.

The fluorescence imaging study was performed with a pulse amplitude modulated fluorometer (PAM) that was equipped with an Imaging-PAM MSeries from Walz (Effeltrich, Germany). The PAM was fitted with a blue LED-Array Illumination Unit IMAG-MAX/L, operating at a wavelength of 450 nm. Leaves had undergone 15 min of dark adaptation, in order to assure the activation of the acceptor side of the photosynthetic apparatus. The maximum quantum yield of photosystem II (PSII), represented as Fv/Fm, the actual quantum yield of PSII [Y(II)] were determined, and the linear electron transport rate (ETR) was calculated during the analysis. The investigation of chlorophyll-*a* fluorescence quenching was conducted in accordance with the methodologies outlined in the publication by Gondor et al. (2021).

### Gas exchange measurements

Gas exchange assessments were conducted after 7 days of PUT treatment, on the last fully developed leaves of the plants with a Ciras 2 Portable Photosynthesis System (Amesbury, USA) The reference CO_2_ level was set at 380 μL L^−1^, with a light intensity of 250 μmol m^−2^ s^−1^. These gas exchange analyses were conducted under ambient room temperature conditions, and air humidity was maintained at 50 ± 3% in both instances. Parameters such as net photosynthetic activity (A), stomatal conductance (gs), and transpiration (E) were measured during the steady-state phase of photosynthesis (Majláth et al. 2021).

### Determination of the level of lipid peroxidation and H_2_O_2_ content

To assess lipid peroxidation, we followed the procedure outlined by Majláth et al. (2021), which involves the determination of MDA levels. The samples were analysed spectrophotometrically at 532 nm with Shimadzu UV–vis 160A (Shimadzu Corp. Kyoto, Japan), with the subtraction of non-specific absorption at 600 nm. The quantification was carried out utilizing an extinction coefficient of 155 mM^−1^ cm^−1^.

For the determination of H_2_O_2_ content in the samples, we employed the ferrous ammonium sulfate/xylenol orange (FOX-1) method, as described by Gay et al. (1999). This method involved spectrophotometric measurements at 560 nm (Shimadzu UV–vis 160A), utilizing an H_2_O_2_ calibration curve for quantification.

### Enzyme assays

To analyse antioxidant enzyme activity, 0.5 g tissue was homogenized in 2.5 ml Tris–HCl buffer (0.5 M, pH 7.5) containing 3 mM MgCl_2_ and 1 mM EDTA. The measurements were conducted using spectrophotometry (Shimadzu UV–vis 160A), following the methodology described by Pál et al. (2005). The activity of glutathione reductase (GR) (EC 1.6.4.2.) activity was determined at 412 nm according to Smith et al. (1988). The reaction mixture contained 75 mM Na-phosphate buffer (pH 7.5), 0.15 mM diethylenetriamine-pentaacetic acid, 0.75 mM 5,5′-dithiobis (2-nitrobenzoic acid), 0.1 mM NADPH, 0.5 mM oxidized glutathione and 50 ml plant extract in a total volume of 1 ml. The increase in absorbance at 412 nm was monitored. The activity of glutathione-S-transferase (GST) (EC 2.5.1.18.) was measured by following changes in the absorbance at 340 nm in a mixture containing 72.7 mM Na-phosphate buffer (pH 6.5), 3.6 mM reduced glutathione, 1 mM1-chloro-2,4-dinitrobenzene and enzyme extract (Mannervik and Guthenberg 1981). The activity of ascorbate peroxidase (APX) (EC 1.11.1.11.) activity was determined in the presence of 0.2 M Tris buffer (pH 7.8) and 5.625 mM ascorbic acid according to Janda et al. (1999). The reaction was started with 0.042% H_2_O_2_. The decrease in absorbance at 290 nm was monitored. The activities of antioxidant enzymes are expressed in units of nkatal (g^−1^ FW).

### Proline and nitric oxide determination

The quantification of proline content was carried out using the Bates method (1973) with slight modifications, which relies on its reaction with ninhydrin. To summarize, 200 mg plant samples were homogenized in distilled water. After centrifugation at 15,000 rpm for 10 min at 4 °C, 0.5 ml supernatant was combined with 0.25 ml of glacial acetic acid and 0.25 ml of ninhydrin reagent. This mixture was incubated at 96 °C for 30 min, then the chromophore generated was subsequently extracted using 1 ml of toluene, and its absorbance at 518 nm was determined using a Shimadzu 160A.

The measurement of NO was conducted utilizing the Griss reagent method (Invitrogen™ Griess Reagent Kit, for nitrite quantitation, Catalog number: G7921) according to the manufacturer's instruction.

### Diamine oxidase and polyamine oxidase enzyme activities

The method employed by Takács et al. (2016) was used to estimate the enzyme activities of diamine oxidase (DAO, EC 1.4.3.6.) and polyamine oxidase (PAO, EC 1.5.3.3.). Enzyme activity was expressed in nmol ∆^1^-pyrroline min^−1^ g^−1^ FW using an extinction coefficient of 1.86 × 103 mol^−1^ cm^−1^.

### Polyamine analysis

The leaf and root samples were subjected to homogenization in a 2 ml solution of 0.2 N HClO_4_ and subsequently placed on ice for 30 min. The homogenates were centrifuged at 4 °C in a centrifuge for 10 min at 10,000 rpm. The supernatant was utilized for pre-column derivatization using dansyl chloride, as described by Németh et al. (2002). The compounds 1,3 diaminopropane (DAP), PUT, SPD, and SPM were subjected to analysis using a reverse phase Kinetex column (C18, 100 × 2.1 mm, 5 μm, Phenomenex, Torrance, CA, USA) by HPLC. The HPLC system employed for this analysis consisted of a W2690 separation module and a W474 scanning fluorescence detector, with excitation at 340 nm and emission at 515 nm (Waters, Milford, MA, USA).

### Gene expression analysis

To conduct gene expression studies, the third, fully matured leaves and roots of 14-day-old plants were collected and promptly preserved in liquid nitrogen. The procedures for total RNA extraction and cDNA synthesis were conducted in accordance with the methodology described by Tajti et al. (2021). The RT-qPCR measurements were conducted using a BioRad CFX96 Touch Real-Time Detection System. The experimental setup included the use of 1 µl of fourfold diluted cDNA, 200 nM forward and reverse primers (the primer sequences can be found in Table 1, 2, and 3), 2.5 µl of PCRBIO Mastermix (PCR Biosystem Ltd., London, United Kingdom), and 2.5 µl of molecular grade water. The 2^−ΔΔCt^ method, as described by Livak and Schmittgen (2001), was employed to ascertain the relative transcript levels.

**Table 1:** Primer sequences for RT-qPCR analysis of reference and gene of interest genes in wheat plants

*Ta35497*	Forward	GTGTGTCCCGTGTCGTGTC	131 bp	(Paolacci et al. 2009)
	Reverse	TCCAGCAGCCCAAAGAGTCC		
*TaADC*	Forward	AGGAGGAGGAGCTCGACATT	137 bp	(Gardiner et al. 2010)
	Reverse	GCCGAACTTGCCCTTCTC		
*TaSPDS*	Forward	AGGTATTCAAGGGTGGCGTG	137 bp	(Pál et al. 2018)
	Reverse	TGGGTTCACAGGAGTCAGGA		
*TaapoPA0*	Forward	CCAGCCTCCAGCTCCGCAAC	113 bp	(Xiong et al. 2017)
	Reverse	GCCCAGCTCCTCCACCTCGTC		
*TapxPAO*	Forward	GCTCATAAATCAGCCCAATTCCA	125 bp	(Xiong et al. 2017)
	Reverse	TTCGCCATTTGTTGAGCTCT		
*TaPUT1*	Forward	GGTCTTCTCCCTCTTGCCTT	156 bp	XM_044548016.1
	Reverse	GTGCTGATCGAGTCCCAGTA		
*TaPUT2*	Forward	TTCATCGCCTTCATCAAGCTG	124 bp	XM_044523314.1
	Reverse	TCACCACGACGATCAGGATAG

**Table 2:** Primer sequences for RT-qPCR analysis of reference and gene of interest genes in maize plants

*ZmβTUB*	Forward	CTACCTCACGGCATCTGCTAT	139 bp	NM_001112218.1
	Reverse	AGGAAGGATGGAGAACACCC		
*ZmADC*	Forward	CTAATATGCCCGTATCCACC	167 bp	NM_001365614.1
	Reverse	GGCAATCATCATAAGTCGCAC		
*ZmSPDS*	Forward	CGAAAGAATCAGTGTCAGAACC	152 bp	AY730048.1
	Reverse	GTGCGGTGTCAGCAAAAGC		
*ZmapoPA0*	Forward	GCAAGTACCATGTCCAGGG	148 bp	NM_001111636.2
	Reverse	CGAGGGAACATGGCTGTCA		
*ZmpxPAO*	Forward	TCCTACTCGTGCGACCTG	142 bp	NM_001176693.2
	Reverse	CGATGCCTGACGAGTAAGC		
*ZmPUT1*	Forward	CATCGACAATGCCCTGTACC	190 bp	XM_035959254.1
	Reverse	AGGAAGGATGGAGAACACCC		
*ZmPUT2*	Forward	GGAACACGGCAATAACACGA	105 bp	BT035190.1
	Reverse	GCCCTCCCTTATGCTCTTCA

**Table 3:** Primer sequences for RT-qPCR analysis of reference and gene of interest genes in rice plants

*OsEF1alpha*	Forward	TTTCACTCTTGGTGTGAAGCAGAT	103 bp	(Phule et al. 2018)
	Reverse	GACTTCCTTCAGGATTTCATCGTA		
*OsADC*	Forward	ATCATCCCAATCCAGCGCCT	107 bp	XM_015787552.2
	Reverse	TGCCTCCCGCCGATGAAGT		
*OsSPDS*	Forward	AGAGCATGTGGTTGCATACGC	69 bp	(Do et al. 2013)
	Reverse	GTGCTGATCGAGTCCCAGTA		
*OspxPA03*	Forward	TTTCTATTGCGAAGGCCATTG	100 bp	(Ono et al. 2011)
	Reverse	ATGCGGCACAAATACCACTGA		
*OspxPAO5*	Forward	CATCCAGAGGTACAACAAAACTAT	118 bp	(Ono et al. 2011)
	Reverse	TTCAAACTTGATGATATTTGCTTTAA		
*OsPUT1*	Forward	GGTGGATGAAGTGGTTGAGC	152 bp	XM_015772006.2
	Reverse	ATTCAGCAATGTCAGCACGG		
*OsPUT2*	Forward	ATTGGCATCATGTTCTCCGC	137 bp	XM_015776404.2
	Reverse	ACCACCCTCAGCTTGATGAA		

#### Statistical analysis

The results were the means of at least ten replicates for each treatment for chlorophyll content, five repetitions for chlorophyll-*a* induction and gas exchange parameters, and three replicates for enzyme activity and HPLC analysis. All reactions for gene expression analyses were performed in triplicate using 3 biological and 3 technical repetitions. The data were statistically evaluated using the standard deviation and *t*-test methods. Significance levels were assessed based on the *p*-value, with a threshold of *p* < 0.05 denoted by a single asterisk (*) in the figures. When the difference reached a significance level of *p* < 0.01 or lower, it was indicated by two asterisks (**).

## Results

### Photosynthesis-related parameters

#### Chlorophyll content and chlorophyll-a fluorescence induction analysis

**Fig. 1:**
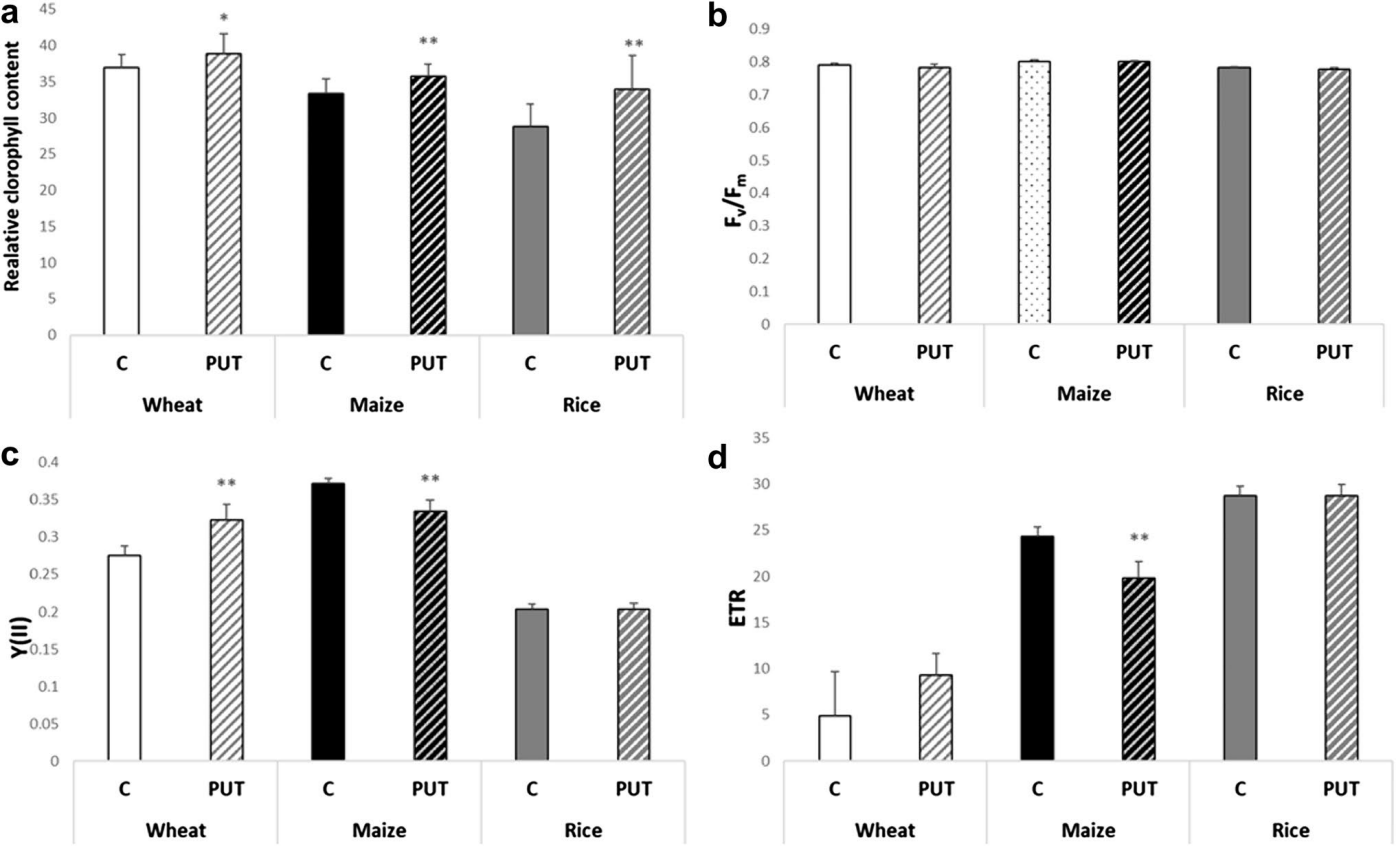
Effects of 7-day 0.3 mM PUT treatments on the relative chlorophyll content (**a**), and chlorophyll-*a* fluorescence induction parameters (**b**: maximum quantum yield of PSII (F_v_/F_m_), c: actual quantum yield of PS II (Y(II)), and d: the electron transport rate (ETR)) in wheat, maize, and rice plants. Data represent mean values ± SD, n = 10. The significant difference at the *p* ≤ 0.05 and *p* ≤ 0.01 level is indicated by * and **, respectively

Application of a 0.3 mM PUT treatment elicited a noteworthy outcome across all three crop plant species, demonstrating a significant increase in leaf chlorophyll content (Fig. 1a).

Analysis of chlorophyll-*a* fluorescence quenching revealed that the PUT treatment did not have a significant impact on the maximum quantum yield of PS II (as indicated by the Fv/Fm parameter) in either examined plant genotypes (Fig. 1b). However, it did influence the photosynthetic activity of PS II, leading to notable differences in both the Y(II) (Fig. 1c) and the ETR (Fig. 1d). Nevertheless, the parameter Y(II) exhibited distinct responses in the three plant species (Fig. 3b). Specifically, in wheat plants, there was a significant increase in the Y(II) value, while it notably decreased in maize plants. No significant changes were observed in rice plants. Following PUT treatment, the ETR value exhibited an increase in wheat. However, no statistically significant alterations were observed in rice and maize (Fig. 1c).

#### Gas exchange measurements

The values of the gas exchange parameters after PUT treatment for the three plant species are shown in Table 4. Notably, there were no substantial alterations observed in any of the plant species as a direct outcome of PUT pre-treatment. The only parameters that displayed significant changes were the transpiration rate (E) in rice.

**Table 4:** Effect of 7-day 0.3 mM PUT treatment on gas exchange parameters (A: net photosynthetic activity, gs: stomatal conductance, and E: transpiration)

		Net photosynthetic activity (A) (μmol CO_2_ m^−2^ s^−1^)	Stomatal conductance (gs) (μmol H_2_O m^−2^ s^−1^)	Transpiration (E) (μmol H_2_O m^−2^ s^−1^)
Wheat	C	12.5 ± 0.55	89.6 ± 3.78	1.40 ± 0.10
	PUT	13.76 ± 1.01	106 ± 17.43	1.20 ± 0.10
Maize	C	12.86 ± 1.21	52 ± 5.56	0.66 ± 0.06
	PUT	13.06 ± 1.27	48 ± 1.52	0.63 ± 0.06
Rice	C	12.52 ± 1.65	95 ± 7.76	1.05 ± 0.10
	PUT	11.62 ± 1.20	89 ± 7.72	0.88 ± 0.08*

The significant difference at the *p* ≤ 0.05 is indicated by *, compared to the adequate control

### Effects of PUT treatment on certain stress markers

#### Lipid peroxidation and H_2_O_2_ content

**Fig. 2:**
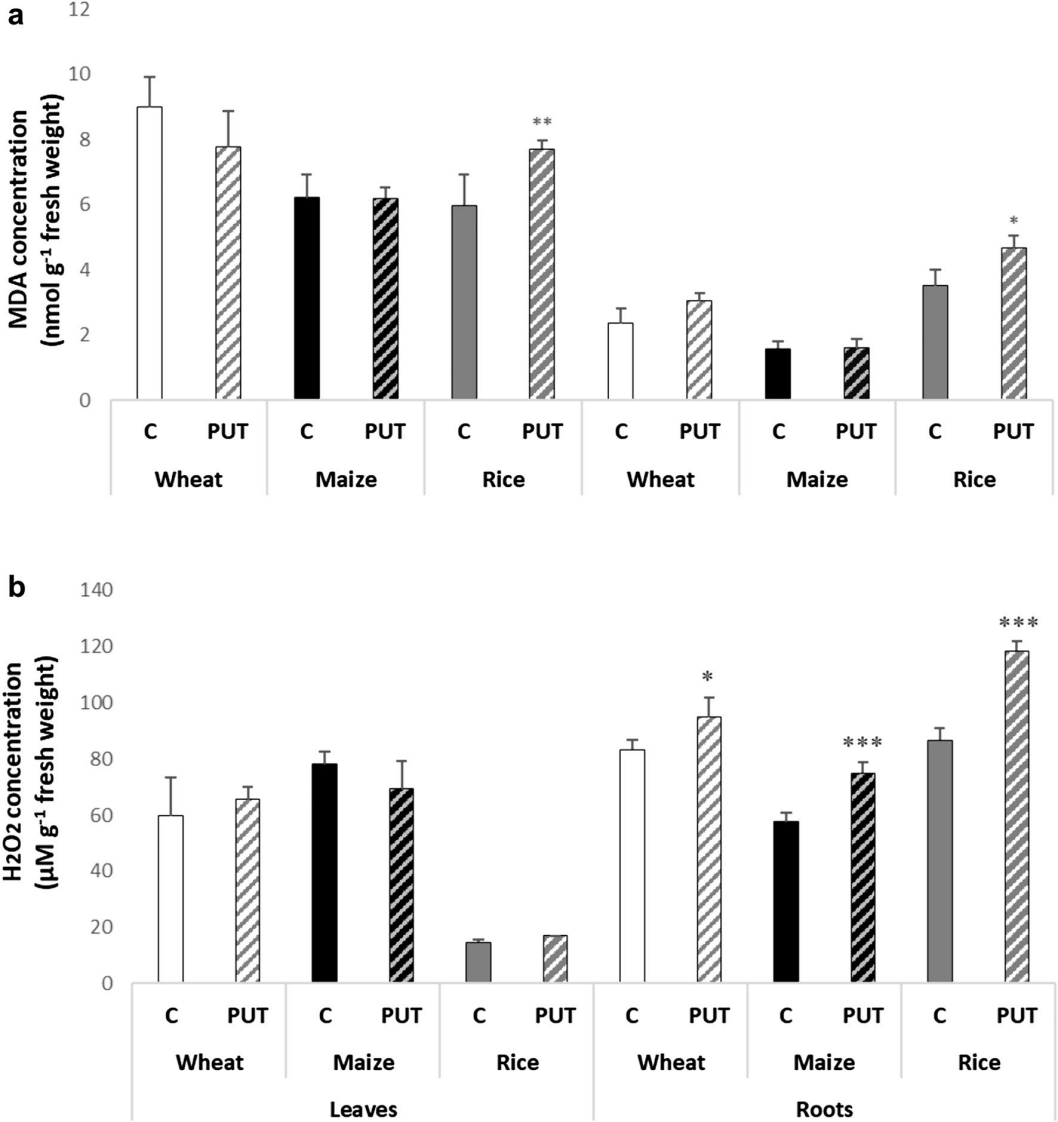
Effects of 7-day 0.3 mM PUT treatments on the MDA concentration (**a**) and H_2_O_2_ concentration (**b**) in the leaves and roots of wheat, maize, and rice plants. Data represent mean values ± SD, n = 10. The significant difference at the *p* ≤ 0.05 and *p* ≤ 0.01 level is indicated by * and **, respectively

The MDA concentration was used to examine lipid peroxidation. PUT treatment did not induce lipid peroxidation in the leaves and roots of the wheat and maize plants, but it did elicit a statistically significant effect in the accumulation of MDA in the rice leaves and roots (Fig. 2a), indicating a condition of stress in the rice plants.

In the leaves of all three crop plants, PUT application had no significant effect on H_2_O_2_ content, but a substantial increase in H_2_O_2_ accumulation was observed in the roots of all three plant species, with the most pronounced effect found in the roots of rice (Fig. 2b).

#### Proline and NO contents

**Fig. 3:**
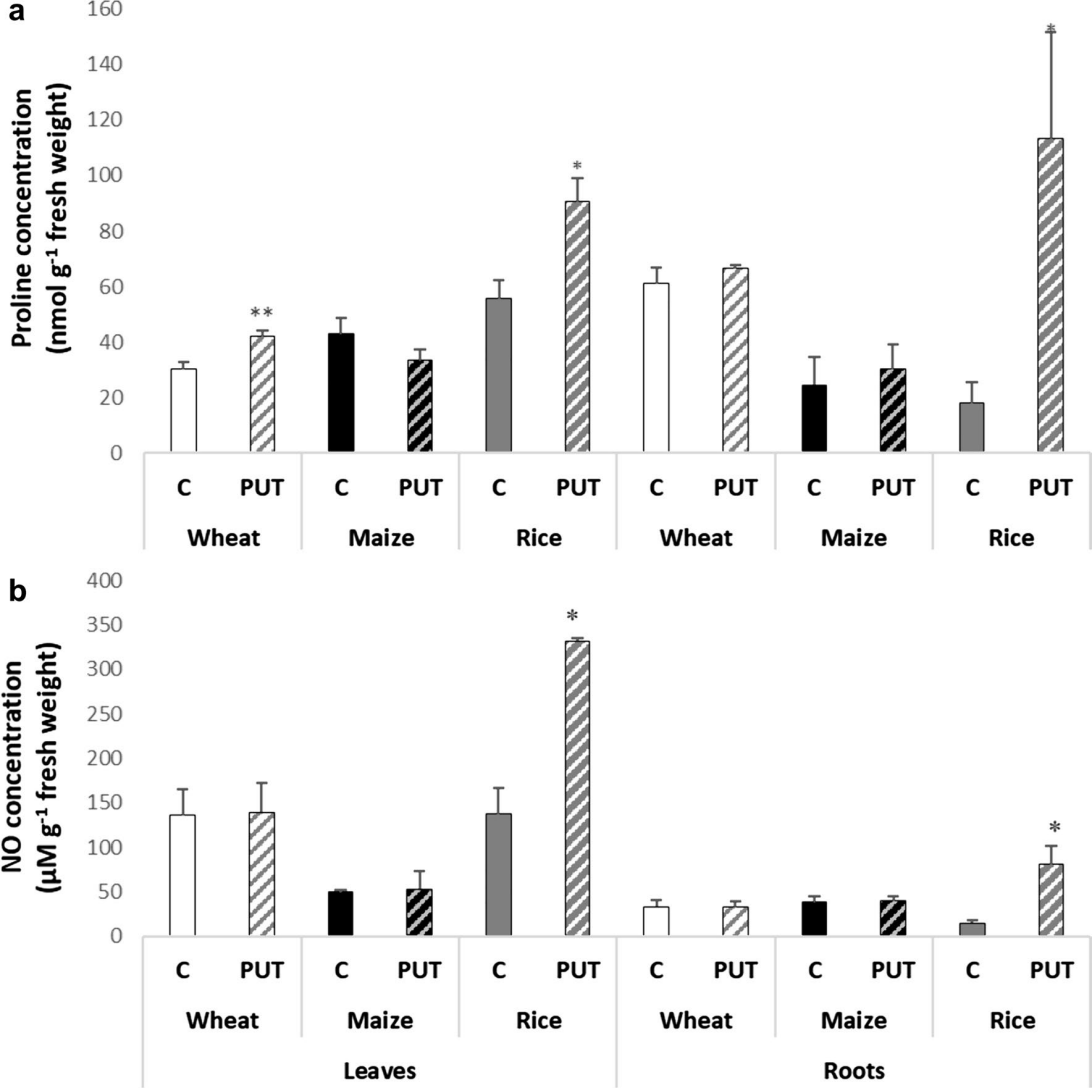
Effects of 7-day 0.3 mM PUT treatments on the proline concentration (**a**) and NO concentration (**b**) in the leaves and roots of wheat, maize, and rice plants. Data represent mean values ± SD, n = 10. The significant difference at the *p* ≤ 0.05 and *p* ≤ 0.01 level is indicated by * and **, respectively

Under the present conditions, as a result of PUT treatment, the level of proline increased slightly, but statistically significantly in the leaves of wheat and rice, while in the roots of rice dramatic proline accumulation was detected compared to the control. Whereas proline levels did not change either in the leaves or in the roots of maize (Fig. 3a). The highest accumulation of proline in the root of the rice plant is also indicative of a stressed condition.

Treatment with 0.3 mM PUT for 7 days did not induce any changes in NO content in the leaves and roots of wheat and maize plants. However, exogenous PUT induced an increase in multiple folds of NO concentration in the leaves and roots of rice plants (Fig. 3b).

#### Antioxidant enzyme activities

**Fig. 4:**
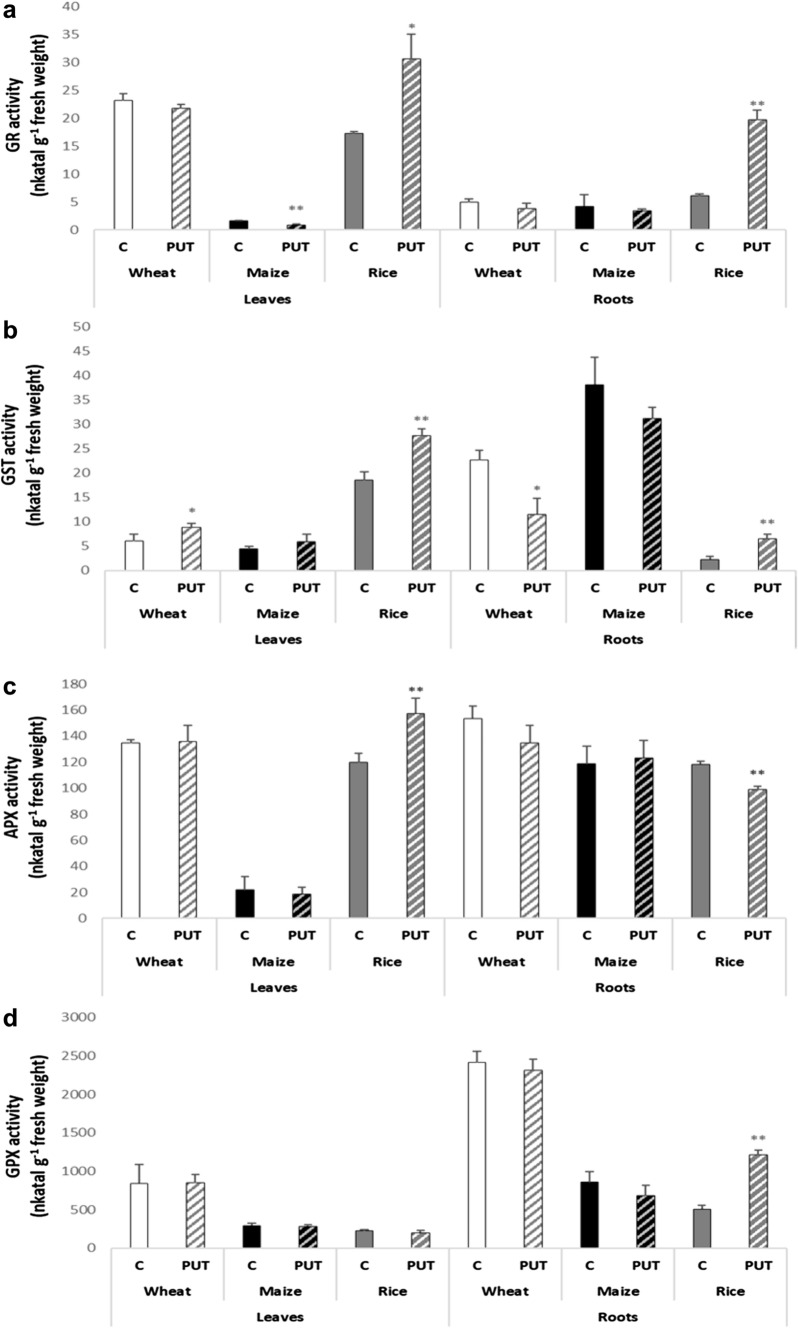
Effects of 7-day 0.3 mM PUT treatments on the **a** glutathione reductase (GR), **b** glutathione-S-transferase (GST), **c** ascorbate peroxidase (APX), and **d** guaiacol peroxidase (GPX) enzyme activity in the leaves and in the leaves and roots of wheat, maize and rice plants. Data represent mean values ± SD, n = 10. The significant difference at the *p* ≤ 0.05 and *p* ≤ 0.01 level is indicated by * and **, respectively

Figure 4 shows that the most remarkable changes were observed again in rice as a result of the PUT treatment. The activity of GR increased in both the leaf and the root of the rice plant. On the contrary, its activity in maize leaves slightly declined after PUT treatment (Fig. 4a). For GST, a significant increase in enzyme activity was also observed in rice plants following PUT treatment both in the leaves and roots (Fig. 4b). Interestingly in wheat plants, PUT treatment increased GST activity in the leaves, but decreased it in the roots (Fig. 4b). PUT treatment caused substantial changes in the activity of APX and GPX also in rice plants. The activity of APX increased in the leaves but decreased in the roots (Fig. 4c) whereas the activity of GPX increased in the roots compared to the control (Fig. 4d).

### Effects of the putrescine treatments on the polyamine metabolism

#### Changes in polyamine contents

**Fig. 5:**
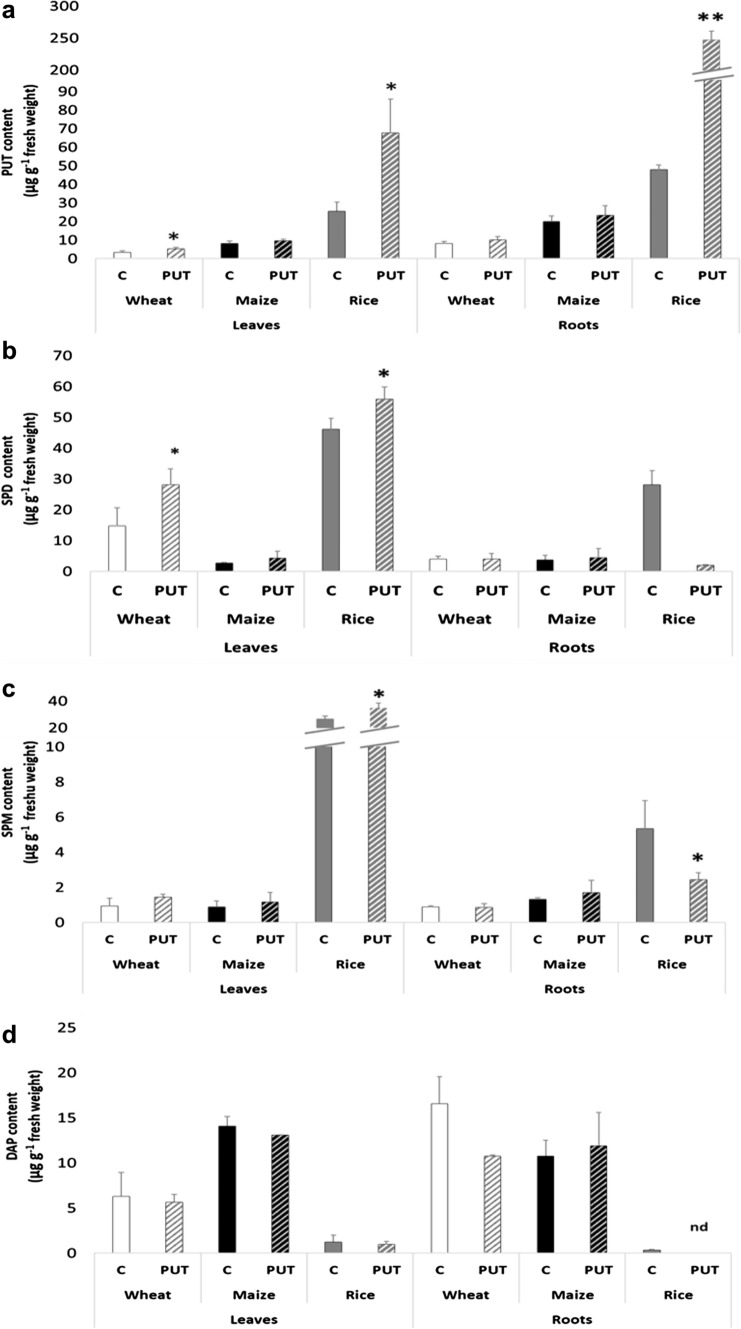
Effects of 7-day 0.3 mM PUT treatments on the PUT (**a**), SPD (**b**), SPM (**c**), and DAP (**d**) contents in the leaves and roots of wheat, Maize, and Rice plants. Data represent mean values ± SD, n = 3. The significant difference at the *p* ≤ 0.05 and *p* ≤ 0.01 level is indicated by * and **, respectively. nd means not detected

Considerable variations were found in the initial PA composition among plant species. Rice plants displayed the highest total PA content in both leaves and roots, compared to wheat and maize. Wheat exhibited the highest levels of SPD, followed by PUT and SPM in leaves, while in the roots PUT content was higher than SPD. In maize leaves the highest PUT content was followed by SPD and SPM, mirroring the pattern observed in maize roots, too. In rice leaves, the PA distribution followed the sequence SPD > PUT ≥ SPM, whereas in roots, it was PUT > SPD > SPM (Fig. 5a–c). Notably, DAP content, which is the catabolite product of terminal oxidation of SPD and SPM, also showed remarkable differences between the plant species, and compared to wheat and maize, in rice, it was very low (Fig. 5d).

While maize plants did not display notable alterations in PUT, SPD, and SPM content following treatment, wheat and especially rice plants exhibited significant changes after PUT treatment (Fig. 5a–d). The content of PUT increased significantly in rice leaves and roots following treatment, in addition in wheat leaves, while no significant differences were observed in case of maize (Fig. 5a). SPD also increased by exogenous PUT in wheat leaves and rice leaves, but decreased in rice roots (Fig. 5b). The changes in SPM level in rice leaves and roots where similar to those described for SPD content (Fig. 5c). In contrast, DAP content remained relatively stable across the three plant species, except for a decrease below the detection limit in rice roots following PUT treatment (Fig. 5d).

#### Activity of PAO and DAO enzymes responsible for terminal catabolism of PAs

The activity of apoplastic PAO and DAO, which are responsible for terminal degradation of SPD/SPM and PUT, respectively. Notably, an increase in PAO activity was observed in the root samples of PUT-treated maize and rice plants (Table 5). While DAO did not exhibit significant changes following PUT treatment in either plant species, whether in leaves or root samples (Table 5).

**Table 5:** Effect of 0.3 mM PUT treatment on the diamine oxidase (DAO) and polyamine oxidase (PAO) enzyme activities after 7 days in wheat, maize, and rice plants

		PAO activity (nkatal g^−1^ FW)		DAO activity (nkatal g^−1^ FW)	
		Leaves	Root	Leaves	Root
Wheat	C	10.53 ± 3.17	13.55 ± 4.97	5.91 ± 1.32	9.44 ± 1.71
	PUT	11.67 ± 1.76	19.40 ± 2.97	6.55 ± 1.20	7.97 ± 0.39
Maize	C	50.42 ± 2.88	23.62 ± 3.02	7.58 ± 0.25	15.70 ± 0.46
	PUT	41.29 ± 6.45	31.30 ± 1.87*	9.09 ± 1.91	14.38 ± 1.19
Rice	C	12.35 ± 0.37	13.64 ± 0.84	8.35 ± 0.09	11.69 ± 1.53
	PUT	12.95 ± 1.75	17.96 ± 1.95*	7.89 ± 0.36	22.89 ± 5.85

Data represent mean values ± SD, n = 3. The significant difference at the *p* ≤ 0.05 level is indicated by *, compared to the adequate control

#### Expression level of certain polyamine metabolism-related genes

**Fig. 6:**
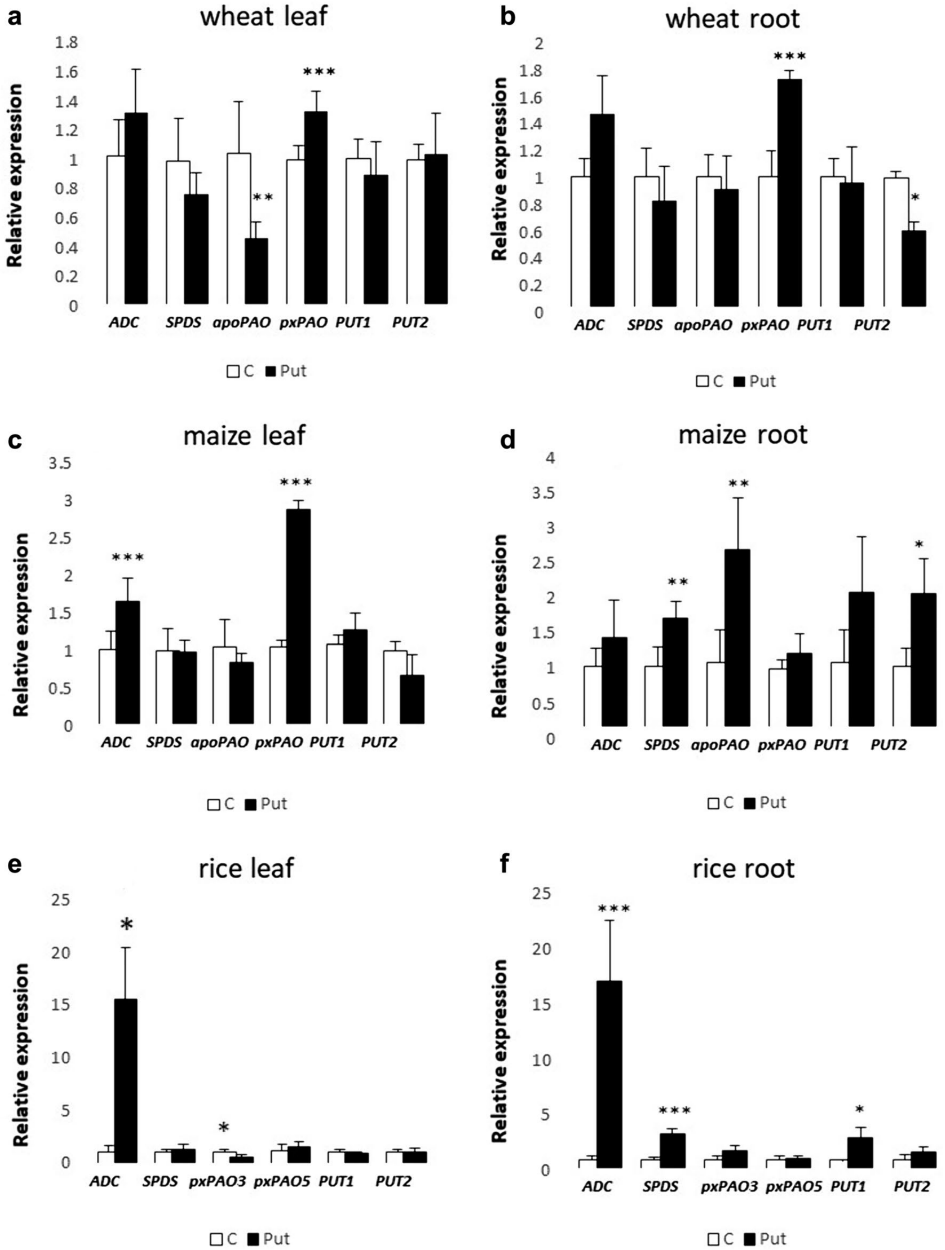
Effects of 7-day 0.3 mM PUT treatments on the expression levels of PA synthesis-related genes, namely arginine decarboxylase (*ADC*) (**a**–**f**) spermidine synthase (*SPDS*) (**a**–**f**), PA metabolism-related genes, namely PA oxidases (**a**–**f**) *apoPAO* and*, pxPAO,* and PA uptake transporter genes namely (**a**–**f**) *PUT1* and *PUT2* in the leaves and roots of wheat, Maize and Rice plants. Data represent mean values ± SD, n = 5. The significant difference at the *P* ≤ 0.05 and *P* ≤ 0.01 level is indicated by * and **, respectively

The application of PUT treatment resulted in statistically significant and distinct expression patterns of certain genes related to PA metabolism (Fig. 6a–f). Substantial increases were detected in the transcript levels of the *ADC* gene in the leaves and roots of rice plants after PUT application (Fig. 6a–b). Regarding *SPDS*, its transcript level is upregulated in maize and rice roots (Fig. 6d, f).

Interestingly, the expression level of the gene encoding the peroxisomal localised PAO (*pxPAO)*, which is responsible for the back-conversion of higher PAs, exhibited a significant increase in the leaves of both wheat and maize plants, in addition in the roots of wheat (Fig. 6a–c). While the transcript level of *PAO*, which encodes the *apoPAO* catalysing the terminal oxidation of SPD/SPM, decreased in wheat leaves, but increased in maize roots (Fig. 6a, c). In contrast, the expression levels of *apoPAO* and px*PAO3* genes showed a notable decrease in all cases, except in maize and rice roots. Notably, PUT treatment had no discernible impact on the expression level of the px*PAO5* gene in either the leaves or roots of rice plants (Fig. 6e–f). In the leaves of all three crop plants, no substantial changes were observed in the gene expression levels of *PUT1* and *PUT2*. However, a significant increase in the expression levels of *PUT2* or *PUT1 genes* was noted in maize and rice roots, respectively (Fig. 6d, f). While in wheat root, *PUT2* expression was inhibited by exogenous PUT (Fig. 6b, d, f).

## Discussion

Although in several cases there is a positive correlation between PA levels and plant growth or stress tolerance, it has become apparent in recent years that not only PA depletion but also excessive PA accumulation may be detrimental (Iannone et al. 2013; Jiménez-Bremont et al. 2014; Szalai et al. 2017). Several authors have demonstrated the bio-stimulant effects of PA application during plant development (Chen et al. 2019) and the ameliorative function of PA treatments against diverse stress factors (Minocha et al. 2014; Li et al. 2015a, b). Nonetheless, the positive effect may vary depending on the investigated plant genotypes, the mode of application, or the concentration of the applied PAs (Szalai et al. 2017; Tajti et al. 2018; Pál et al. 2019). Thus, it remains a pertinent question: is more always better when it comes to PAs? Only a few investigations have focused on the negative effects of PAs up to the present. PA treatment has been reported to lead to root growth inhibition and alterations in plant morphology in *Arabidopsis* (Tassoni et al. 2000). In maize it induced programmed cell death (PCD) (Tisi et al. 2011), due to the cytotoxic by-products of PA metabolism (Moschou and Roubelakis-Angelakis 2014). Prior research also indicated a negative effect of 0.5 mM PUT treatment during cadmium stress, while the inhibition of PUT synthesis was favourable in rice (Pál et al. 2017). In maize 0.5 mM PUT pre-treatment did not result in a pronounced protective effect against osmotic stress as it was found in wheat due to the higher PA accumulation (Szalai et al. 2017). So, PAs seem to play important roles in normal cellular functions, plant development, or stress tolerance, but the balanced PA metabolism achieved by the regulation of biosynthesis, back-conversion, catabolism, and conjugation is the most important factor during the outcome of their effects (Handa et al. 2018). In the present study in the same vein, the potential effects of 0.3 mM PUT treatment on three economically important plant species, namely wheat, maize, and rice were tested, in order to reveal the changes in PA metabolism in the background, and their responsibility for the observed differences.

PAs can exert their effects on photosynthesis at several levels. PA treatments protected the chloroplast ultrastructure by preserving the thylakoid membrane structure, and could improve the photosynthetic capacity by increasing the level of the photochemical efficiency of PSII, interacting directly with thylakoid membranes, thus decreasing the loss of LHCII, increasing chlorophyll content, influenced stomatal opening, improved the leaf CO_2_ assimilation rate (Shu et al. 2012a, b; Najafpour 2012; ElSayed et al. 2022; González-Hernández et al. 2022). Navakoudis et al. (2007) found that PUT can directly increase the size of the LHCII antenna complex, and bind to the PSI and PSII core proteins. Consequently, increased electron transport rate and photosynthetic activity can be attributed to PUT treatment. Our results demonstrate that treatment with 0.3 mM PUT significantly increased the chlorophyll content of the leaves of all three plant species, indicating a beneficial effect on photosynthetic processes to a certain extent. However, chlorophyll-*a* fluorescence quenching analyses revealed that PUT treatment increased the actual quantum yield (Y(II)) only in wheat, did not influence maize, and decreased it in rice plants. At the same time, these changes were not accompanied by changes in CO_2_ exchange parameters. PUT treatment only induced a slight decrease in the transpiration rate in rice.

Although, under the given conditions 0.3 mM PUT treatment could not induce pronounced changes and differences in the photosynthesis-related parameters, the determination of certain stress markers proved that PUT application was not beneficial for all the three plant species. PUT treatment did not induce lipid peroxidation or H_2_O_2_ accumulation in the leaves of wheat and maize. Nonetheless, a statistically significant increase was observed in the level of MDA and H_2_O_2_ in the roots of all three crop plants, indicating that the roots were subjected to oxidative stress conditions. In addition, in the leaves of rice increased MDA content was detected revealing that rice is more sensitive to exogenous PUT. Results also suggested that not the decreased photosynthesis activity may be responsible for ROS production, the other processes. As both the terminal catabolism and the back-conversion of the excess PA produce H_2_O_2_ PA metabolism can be implicated.

Further analysis of other stress markers, namely proline and NO contents, confirmed this hypothesis. Dramatic accumulation of both compounds was observed after PUT treatment in rice leaves and roots. Proline is an essential amino acid with multiple roles in plants. It functions as a nitrogen source, stress indicator, osmolyte, and antioxidant molecule in plants (Majumdar et al. 2016; Razavizadeh et al. 2017), thus the increase in proline content due to PA treatment indicated its essential protective role in rice roots under stress conditions. NO is a crucial gaseous free radical in plants, acting as an intra- and intercellular messenger to trigger processes including defence-related gene expression, programmed cell death, stomatal closure, seed germination, and root development (Neill et al. 2003; Lamotte et al. 2004). NO production can be mediated by H_2_O_2_ resulting from the oxidation of PAs via DAO and PAO enzymes, or through other unidentified mechanisms associated with the PAs pathway (Wimalasekera et al. 2011). It is worth mentioning that proline and PA synthesis use a common precursor (glutamate), in addition, the catabolism of PAs may also be involved in proline production (Su and Bai 2008), furthermore, NO production is intricately linked to PA metabolism (Flores et al. 2008). Thus the increased level of these compounds can not only indicate stress condition in rice, but reflect on the imbalance in PA metabolism.

Along with these changes, induced activities of leaf and root GR, leaf and root GST, leaf APX, and root GPX were found in rice plants, indicating that the activated antioxidant system tried to maintain the redox balance. Species-specific role of PAs treatments in modulating the antioxidant defence system has been reported in various cases (Shao et al. 2022). PAs can generally activate antioxidant enzymes and modulate ROS homeostasis and oxidative damage by inhibiting H_2_O_2_ accumulation (Singh-Gill & Tuteja 2010). However, under the present conditions, the induction of the antioxidant system in rice plants indicated again the disturbance of PA homeostasis.

To highlight the role of the PA pool in the above-described stress conditions in rice, detailed analyses of PA metabolism were performed. Plants use a variety of mechanisms to control endogenous PA levels, such as the synthesis of PUT, its further synthesis to higher PAs, conjugation of them to small molecules, conversion of higher PAs back to PUT in the PA cycle, and terminal oxidation of them (Pál et al. 2015). Although upon exposure to exogenous PA, other mechanisms may be also involved, like modulation of PA uptake, and translocation from the roots to the shoots. PUT application induced a slight increase in PUT and SPM contents of wheat leaves, did not influence the DAP content and DAO or PAO activities, in addition, did not induce characteristic changes in the expression level of PA metabolism-related genes, except slight *pxPAO* induction both in the leaves and roots, and *PAO* inhibition in the leaves. These findings revealed that in wheat plants the applied PUT treatment did not affect the PA metabolism or the plants can re-adjust it properly, in order the maintain PA homeostasis. In maize, more changes were detected. Although the PA contents were not affected, in the roots the PAO activity increased, furthermore at the gene expression level, *PAO* was also induced in the roots. In contrast, in maize leaves the expression level of *pxPAO* increased after PUT treatment. These changes reflect that excess PUT induced the PA cycle and catabolism both at enzymatic and gene expression levels, which in turn helps the maize plants in acclimation to changed conditions. At the same time, PUT treatment caused pronounced PUT accumulation in the leaves and roots of rice, indicating not only the uptake of PUT but also its translocation. The application of PUT induced the accumulation of higher PAs (SPD or SPM) in the leaves of rice plants, indicating that PUT treatment resulted in additional synthesis of SPD or SPM. However, interestingly the *ADC* gene expression also increased both in the leaves and roots, which proved that in vivo PUT synthesis was also induced, and responsible for the dramatic PUT accumulation. In the roots of rice, the amount of SPD and SPM decreased after PUT treatment despite the increased transcript level of *SPDS*, but partly due to the increased PAO activity. In this instance, however, there was no detectable DAP accumulation following PUT treatment. Notably, even under controlled conditions, rice had the lowest DAP.

## Conclusion

PAs, including PUT, have been recognized for a long time to play essential roles in cellular growth, differentiation, and stress responses. While PUT had beneficial effects on certain aspects of plant physiology, its impact varied depending on the plant species and its inherent capacity to regulate PA homeostasis. This study challenges the simplistic notion that the higher PA level is always the better, emphasizing the context-dependent responses of plants to PA treatments. Wheat, maize, and rice were positively affected by PUT treatment in terms of chlorophyll content, but an investigation of various stress markers testified that rice plants experienced oxidative stress. As in rice, the initial PA content was much higher than in wheat or maize, disruption in PA metabolism after PUT application could be responsible for the observed negative effects. In conclusion, while PUT has the potential to improve plant growth, development, and stress tolerance, its negative effects vary across plant species, highlighting the importance of the dynamic nature of the PA metabolism. To fully understand the underlying mechanisms and maximize the potential use of PUT for crop improvement, additional research is required.
